# The physical activity paradox in relation to work ability and health-related productivity loss in Korea

**DOI:** 10.4178/epih.e2023096

**Published:** 2023-10-28

**Authors:** Heejoo Ko, Dohwan Kim, Seong-Sik Cho, Mo-Yeol Kang

**Affiliations:** 1 College of Medicine, The Catholic University of Korea, Seoul, Korea; 2 Department of Occupational and Environmental Medicine, Dong-A University College of Medicine, Busan, Korea; 3 Department of Occupational and Environmental Medicine, Seoul St. Mary’s Hospital, College of Medicine, The Catholic University of Korea, Seoul, Korea

**Keywords:** Exercise, Efficiency, Work ability, Health-related productivity loss

## Abstract

**OBJECTIVES:**

The physical activity paradox suggests that occupational physical activity (OPA), unlike leisure-time physical activity (LTPA), may detrimentally impact health. We explored the relationships of OPA and LTPA with work ability (WA) and health-related productivity loss (HRPL).

**METHODS:**

This study included 5,501 workers in Korea who were recruited in 2021 through a web-based cross-sectional questionnaire. The questionnaire was utilized to quantify OPA and LTPA in metabolic equivalents, while WA and HRPL were also measured. Non-parametric regression, using a generalized additive model (GAM), was employed to visualize the relationships of LTPA and OPA with WA and HRPL. Mean differences in WA and HRPL, in relation to OPA and LTPA, were examined using linear regression models. These models were adjusted for covariates including sex, age, body mass index, education level, alcohol consumption, smoking history, insomnia, occupation, hours worked, and income.

**RESULTS:**

The GAM and linear regression analyses revealed that higher LTPA corresponded with higher WA and lower HRPL. In contrast, as OPA increased, WA decreased and HRPL increased. However, within the group with high OPA, HRPL was not significantly lower in the high-LTPA subgroup relative to the low-LTPA subgroup (mean difference=1.92%, p=0.343). This pattern was especially pronounced among workers aged 60 years and older, with an increase in HRPL observed with increasing LTPA among the respondents with high OPA.

**CONCLUSIONS:**

High LTPA levels were associated with elevated WA and diminished HRPL. In contrast, higher levels of OPA were associated with lower WA and higher HRPL.

## GRAPHICAL ABSTRACT

[Fig f4-epih-45-e2023096]

## INTRODUCTION

Health is frequently considered an individual’s most valuable asset. When health is less than optimal, basic daily activities, including the ability to work, can be severely compromised. Under the theoretical framework of the human capital model, a person’s work ability (WA) and workplace productivity are directly proportional to that individual’s health status [1]. Therefore, promoting personal health is crucial to maintaining WA and enhancing labor productivity among employees. Examples of personal health promotion include the adoption of healthy lifestyle behaviors, such as engaging in regular exercise.

Regular physical activity can yield substantial health benefits, including the prevention of conditions such as cardiovascular disease, diabetes, cancer, and osteoporosis. It also may have a positive impact on mental health [2–4]. However, not all physical activity contributes favorably to health. The physical activity paradox is a phenomenon in which occupational physical activity (OPA) can have detrimental effects on health, in contrast to the beneficial impacts of leisure-time physical activity (LTPA). This phenomenon suggests that LTPA and OPA should be considered separately. For instance, while higher LTPA is associated with a lower prevalence of cardiovascular disease or diabetes, elevated OPA is correlated with a higher prevalence of these diseases [5,6].

This pattern may also be evident in labor market performance. For instance, a higher level of OPA is linked with an increased likelihood of experiencing burnout at work [7]. Conversely, a higher level of LTPA is associated with increased WA [8]. A study examining long-term sickness absence and physical activity found that higher OPA corresponded to an elevated risk of such absence, while higher LTPA was associated with reduced risk, thus demonstrating the physical activity paradox [9]. Consequently, the influence of LTPA and OPA on labor market performance can be complex and seemingly contradictory. Therefore, the paradoxical associations of LTPA and OPA with WA and health-related productivity loss (HRPL) constitute an important area of research, key to improving our understanding of these intricate relationships and developing evidence-based interventions to promote improved health and productivity in the workplace. Such research can benefit workers, employers, policymakers, and society at large by contributing to a healthier and more productive workforce.

However, research on the paradoxical associations of LTPA and OPA with WA and/or HRPL has been limited, although reports have described the physical activity health paradox in the context of various health outcomes [10]. Therefore, the present study was conducted to explore the physical activity paradox in relation to WA and HRPL. Since these issues are relevant to aging workers, we also investigated whether effect modifications were present depending on age group. The findings from this study could offer a scientific foundation for practical guidance to help workers maintain productivity and WA, as well as enhance their overall health.

## MATERIALS AND METHODS

### Study participants

We used the initial dataset from a survey known as the Korean Work, Sleep, and Health Study, an ongoing nationwide panel study initiated in 2022. Participants were recruited in July 2022 via the online survey platform EMBRAIN. In brief, panelists were invited to participate in the survey through a process of random sampling, which was stratified by sex, age, and occupation. The initial screening process was completed by a total of 5,517 participants, all of whom were wage earners of varying occupations and aged 19 years or older. Inclusion in the study was contingent upon participants providing complete responses to questions designed to gather the necessary information, including sex, age, body mass index (BMI), education level, occupation, hours worked, income, alcohol consumption, smoking status, and insomnia severity index, along with questions used to calculate physical activity, WA, and HRPL. Consequently, 16 individuals who did not provide complete answers to the questions required to determine HRPL were excluded from the study. Ultimately, a total of 5,501 participants were enrolled.

### Independent variables: occupational physical activity and leisure-time physical activity

The Global Physical Activity Questionnaire (GPAQ) [11,12] was employed to measure OPA and LTPA. Its purpose was to collect data on an individual’s levels of physical activity in both work and leisure contexts. The questionnaire consists of 12 questions about the intensity, frequency, and duration of both vigorous and moderate physical activity. These questions encompass activities undertaken during work and leisure time. The reliability and concurrent validity of the GPAQ have been primarily documented in adult populations from Asia and Europe, as evidenced by 20 publications [13].

Metabolic equivalents (METs) were used to quantify the intensity of physical activity. METs reflect the ratio of an individual’s metabolic rate during physical activity compared to that person’s metabolic rate at rest. A single MET is defined as the energy expenditure of sitting quietly, which equates to a caloric consumption of 1 kcal/kg/hr. Moderate and vigorous activities were assigned values of 4 and 8 METs, respectively. The survey questions were used to separately calculate OPA and LTPA in MET-min/wk. If certain activities were not performed, such as vigorous-intensity sports, the corresponding values were considered to be 0. To calculate LTPA, the following formula was used: (days engaged in vigorous-intensity sports×minutes spent×8)+(days engaged in moderate-intensity sports×minutes spent×4). Similarly, OPA was computed using the following formula: (days involving vigorous-intensity activity at work×minutes spent×8)+(days involving moderate-intensity activity at work×minutes spent×4) [11].

High physical activity was defined as that exceeding 600 MET-min/wk. This is equivalent to 150 minutes of moderate-intensity and 75 minutes of vigorous-intensity physical activity, or a comparable combination, for both OPA and LTPA. The criterion of 600 MET-min/wk was derived from the World Health Organization recommendations on physical activity for health, as outlined in the GPAQ questionnaire guidelines [11].

### Dependent variables: work ability and health-related productivity loss

WA was evaluated using the Work Ability Index (WAI) [14,15], a tool frequently employed in clinical occupational health research. The WAI has been utilized in numerous countries and has demonstrated high reliability within the Korean context [16]. The questionnaire used in this study assessed various facets of WA, health status, and mental well-being and was divided into 7 sections, each containing specific questions related to WA. Participants were asked to rate their current WA, assess their WA in relation to job demands, identify any existing diseases and diagnoses, estimate work impairment, report any days taken off work due to illness, predict their WA for the next 2 years, and provide reflections on their mental capacities.

To compute the total score, the points from each section were summed, with specific items receiving additional consideration. Factors such as work demand, with potential options including physically demanding, mentally demanding, or both, were considered. In terms of current diseases, the scoring system incorporated only diagnoses confirmed by physicians.

The scores can be tallied to yield a range from a minimum of 7 points to a maximum of 49 points, with higher scores indicating better WA. This score can be utilized to categorize a participant’s WA as poor (7–27 points), moderate (28–36 points), good (37–43 points), or excellent (44–49 points).

HRPL was measured using the Work Productivity and Activity Impairment Questionnaire, a general health version. The reliability and validity of this questionnaire had been previously reported [17]. The Korean version is accessible online ( http://www.reillyassociates.net/WPAI_Translations.html), and the translation process was standardized through independent translations, back-translation, and expert reviews. This questionnaire was designed to evaluate the impact of health on work productivity and daily activities. It included 6 questions about employment status, hours of work missed due to health and other reasons, actual working hours, the effects of health problems on work productivity, and the influence of health issues on regular daily activities. The overall decrease in work productivity, including absenteeism and presenteeism, was determined based on the responses to these questions.

Absenteeism refers to the degree to which workers are absent from work. The productivity loss associated with absenteeism is calculated by determining the percentage of working hours missed due to health-related issues within the preceding 7 days. Presenteeism, in contrast, is defined as being physically present at work but experiencing impairment due to health problems. The productivity loss attributed to presenteeism is calculated by determining the percentage of working hours lost due to health issues during the same 7-day period. The HRPL, expressed as a percentage, is computed by adding the percentages of absenteeism and presenteeism. It signifies the total percentage of work hours lost due to health-related absence and productivity loss over the past 7 days.

### Covariates

Demographic variables such as age and sex, lifestyle behaviors including alcohol consumption and smoking status, education level, occupation, hours worked, income, sleep quality, and BMI were considered as covariates due to their clinical importance and inclusion in previous studies [7–9]. Sleep quality was evaluated using the Insomnia Severity Index (ISI), which ranges from 0 points to 28 points. Higher scores on the ISI indicate more severe insomnia, with 0–7 points suggesting no clinically meaningful insomnia; 8–14 points indicating subthreshold insomnia; 15–21 points representing moderate insomnia; and 22–28 points denoting severe insomnia [18].

### Statistical analysis

Participants were categorized based on demographic characteristics, and the levels of LTPA, OPA, WA, and HRPL were documented for each demographic group. Given the absence of evidence of linear relationships in prior studies, a non-parametric regression approach using a generalized additive model (GAM) was employed to illustrate the associations between LTPA and WA, LTPA and HRPL, OPA and WA, and OPA and HRPL. Generalized cross-validation scores and thin-plate regression splines were utilized [19]. Adjustments were made for factors including sex, age, BMI, education level, alcohol consumption, smoking status, insomnia, occupation, hours worked, and income. Additionally, the relationships between LTPA and WA, as well as those between LTPA and HRPL, within the high-level and low-level OPA groups were illustrated using the same method.

Linear regression models were employed to investigate the differences in WA and HRPL between the LTPA and OPA groups. Participants were divided into high-activity and low-activity groups based on LTPA, using a cut-off of 600 MET-min/wk, and this was treated as a categorical variable. The same procedure was followed for OPA. Low LTPA and low OPA levels were used as reference points in the regression models. This approach enabled examination of the mean differences and 95% confidence intervals (CIs) of WA and HRPL according to the levels of LTPA and OPA, as well as their combinations. Three linear regression models were utilized: model 1, crude; model 2, adjusted for sex and age; and model 3, adjusted for sex, age, BMI, education level, alcohol consumption, smoking history, insomnia, occupation, hours worked, and income. When applicable, covariates were incorporated for adjustment as continuous variables, including age, BMI, hours worked, and income.

An analysis stratified by age group was conducted to determine whether the results differed for participants older than 60 years. All statistical analyses were performed using R version 4.2.2 (R Foundation for Statistical Computing, Vienna, Austria). A 2-tailed p-value of less than 0.05 was established as the threshold for statistical significance.

### Ethics statement

All participants signed a consent form, and anonymity and confidentiality were ensured. The study protocol was approved by the Institutional Review Board of Dong-A University (IRB No. 2-1040709-AB-N-01-202202-HR-017-06).

## RESULTS

Table 1 presents the levels of physical activity for each demographic subgroup, as well as the distribution of participants within these subgroups. Male workers exhibited higher levels of LTPA and OPA than their female counterparts. However, no significant sex-based disparity was observed in WA or HRPL. When considering occupation, white-collar workers demonstrated the lowest OPA levels. In contrast, pink-collar workers and blue-collar workers exhibited progressively higher OPA levels.

**Figure 1:**
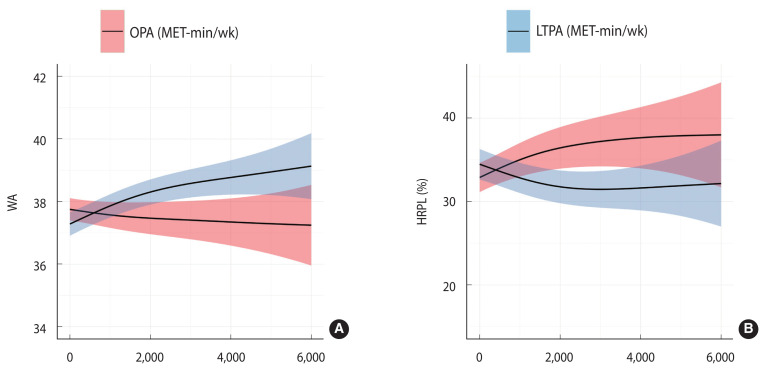
Generalized additive model of (A) work ability (WA) and (B) health-related productivity loss (HRPL) according to occupational physical activity (OPA) and leisure-time physical activity (LTPA). MET, metabolic equivalent.

Figure 1 illustrates the relationships between LTPA and WA, LTPA and HRPL, OPA and WA, and OPA and HRPL, as determined using the GAM. As OPA increased, WA tended to decrease, whereas an increase in LTPA was associated with an increase in WA. HRPL tended to increase with an increase in OPA, but decreased as LTPA increased.

Table 2 presents the results of the linear regression models for WA and HRPL in relation to LTPA and OPA. In the crude model (model 1), the high-LTPA subgroup demonstrated a higher WA (mean difference, 1.50) than the low-LTPA subgroup, while the high-OPA subgroup exhibited a mean difference of −1.20 for WA in relation to the low-OPA subgroup. Models 2 and 3, which were adjusted for covariates, also demonstrated higher WA in the high-LTPA group (mean differences of 1.51 and 1.10 for models 2 and 3, respectively) and lower WA in the low-OPA group (mean differences of −1.39 and −0.90 for models 2 and 3, respectively). In all models, HRPL was lower in the high-LTPA group (mean differences of −4.57, −5.02, and −3.97% for models 1, 2, and 3, respectively), while HRPL was higher in the high-OPA group (mean differences of 6.56, 7.18, and 5.93% for models 1, 2, and 3, respectively). All differences were statistically significant.

**Figure 2:**
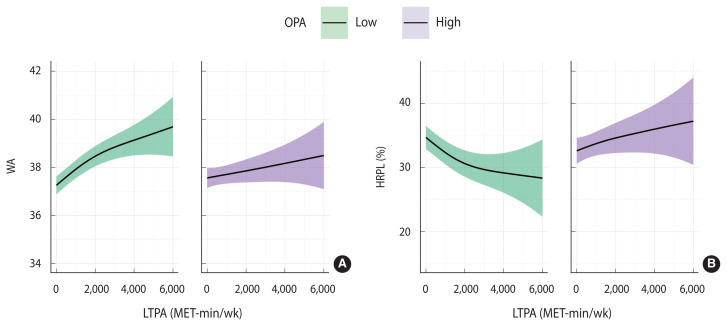
Generalized additive model of leisure-time physical activity (LTPA) according to (A) work ability (WA) and (B) health-related productivity loss (HRPL) within each occupational physical activity (OPA) subgroup. MET, metabolic equivalent.

Figure 2 illustrates the relationship between LTPA and WA, as well as that between LTPA and HRPL, for the low-OPA and high-OPA groups using a GAM adjusted for all covariates. Regardless of the OPA level, WA was observed to increase with increasing LTPA. However, HRPL decreased with increasing LTPA only in the low-OPA group. Conversely, in the high-OPA group, HRPL increased in tandem with LTPA.

Table 3 displays the results of the linear regression models for WA and HRPL, based on the various combinations of OPA and LTPA subgroups. For all models, the highest WA was observed in the group with low OPA and high LTPA, while the lowest was seen in the group with high OPA and low LTPA. These results were statistically significant. In model 3, the group with high OPA and low LTPA exhibited an average WA that was 1.94% (95% CI, 1.28 to 2.60) lower than that of the group with low OPA and high LTPA. Similarly, HRPL was lowest in the group with low OPA and high LTPA across all models, a finding that was statistically significant. This was followed by the groups with low OPA and low LTPA, high OPA and high LTPA, and high OPA and low LTPA, in that order. The group with high OPA and low LTPA exhibited the highest HRPL. In model 3, the group with high OPA and low LTPA showed an average HRPL that was 8.72% (95% CI, 5.51 to 11.93) higher than that of the group with low OPA and high LTPA.

**Figure 3:**
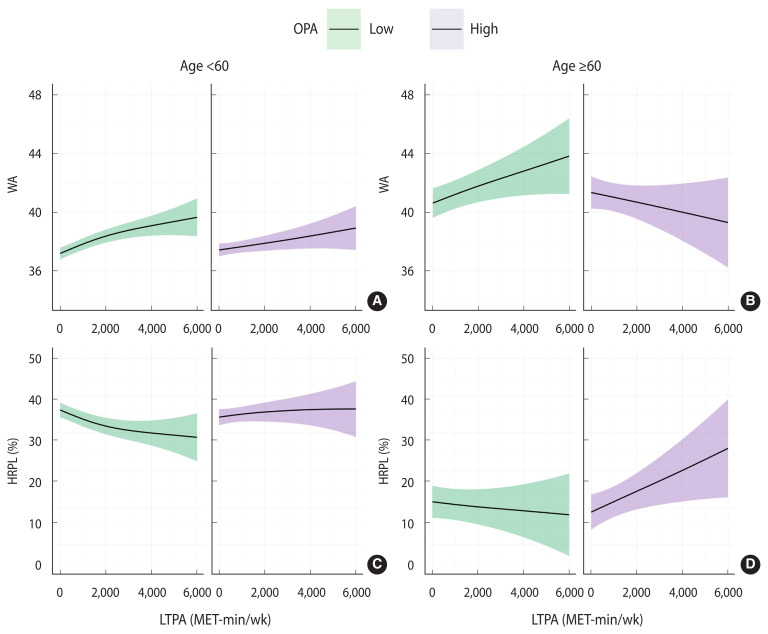
Generalized additive model of leisure-time physical activity (LTPA) according to (A and C) work ability (WA) and (B and D) health-related productivity loss (HRPL) within each occupational physical activity (OPA) subgroup by age group. MET, metabolic equivalent.

A sensitivity analysis was conducted according to age, with participants divided into those younger than 60 years and those 60 years or older. Table 4 presents the outcomes of the linear regression models for WA and HRPL within each age group, categorized by OPA and LTPA subgroups. Figure 3 illustrates the results of the non-parametric regression of LTPA on WA and HRPL within each age group and OPA subgroup. In the under-60 age group, both linear regression and GAM analyses showed trends consistent with those observed in the previous analysis. However, among those 60 years and older, the high-LTPA group did not display significantly lower HRPL in the linear regression model relative to the low-LTPA participants. In contrast, the high-OPA group demonstrated a significant reduction in WA and an increase in HRPL in comparison with the low-OPA group, even among those at least 60 years old. Furthermore, in that ≥60-year-old population, the WA of the high-OPA group was decreased, which contradicted the trend observed in the younger subgroup. As shown in the GAM, HRPL also markedly increased with increasing LTPA, which differed from the pattern observed for the under-60 age group.

## DISCUSSION

In this study, we explored the associations of OPA and LTPA with WA and HRPL among Korean workers. We found that higher LTPA corresponded to higher WA and lower HRPL. In contrast, elevated OPA was associated with decreased WA and increased HRPL. These results align with the physical activity paradox as it pertains to HRPL and WA [20,21].

The findings from our analysis resemble those of previous studies, although drawing direct comparisons may be challenging due to differing work environments and methodologies. Notably, LTPA has been shown to increase WA [8,21]. However, workers assigned to physically demanding tasks have exhibited decreases in WA [22,23]. A systematic review of 31 randomized controlled trials and non-randomized controlled studies examined the impact of workplace nutritional and physical activity interventions on employee productivity, work performance, and WA [24]. Substantial reductions in absenteeism, improvements in job performance, increased WA, and improved productivity have been reported in studies focusing on physical activity interventions (i.e., non-occupational physical exercise) within the workplace or at multiple levels (organizational and individual). In contrast, research indicates that workers in teaching hospitals experience a decrease in productivity and work limitations associated with mechanical workloads, indicating difficulties in performing tasks during a portion of their work time [25]. However, due to the scarcity of studies investigating the effects of OPA on HRPL, definitive conclusions cannot be drawn.

The health condition of employees may serve as a key factor in sustaining or improving WA and productivity in the workforce [26]. WA is determined by the equilibrium between an individual’s physical or mental resources and the work demands experienced by that person [22]. While LTPA can bolster an individual’s resources, it may also indicate high work demands, potentially leading to adverse effects. Similarly, in the context of HRPL, LTPA could enhance the health status of workers, whereas OPA may be detrimental due to the physical demands of work. Consequently, these types of physical activities appear to exert contradictory effects on HRPL.

Notably, however, LTPA does not always yield favorable outcomes. Unlike their younger counterparts, workers over 60 years old did not experience benefits of increased LTPA in the WA and HRPL categories. This pattern was also evident in the high-OPA group. These findings imply that for older adults, excessive exercise may reduce work productivity, particularly for those who already engage in substantial physical activity at work. While LTPA in later life is beneficial for health and reduces the risk of various diseases [27], this may not hold true for those with physically demanding jobs. This observation aligns with the findings of previous research that incorporated coronary heart disease as an outcome variable [28]. When socio-demographic and conventional coronary risk factors were considered, the incidence of coronary events was increased by approximately 4-fold among workers with physically demanding jobs who also participated in moderate-to-vigorous physical exercise during their leisure time. This can be attributed to the fact that the combination of strenuous work and excessive exercise results in prolonged cardiovascular overload [29]. On the other hand, considering the cross-sectional nature of these data, it is important to acknowledge the possibility that poor physical condition or work ability could have contributed to lower levels of LTPA.

The phenomenon wherein LTPA and OPA are associated with differing health outcomes is commonly described as the physical activity health paradox [5]. Holtermann et al. [6] proposed a series of hypotheses to clarify the potential underlying mechanisms of this paradox. The authors suggested that OPA may (1) be of too low intensity or too long in duration, (2) cause an increase in the 24-hour heart rate, (3) lead to an elevation in the 24-hour blood pressure, (4) often be undertaken without adequate recovery time, (5) frequently be performed under conditions of low worker control, and (6) exacerbates inflammation.

Our study, which involved a large database of 5,517 workers, was unique in its exploration of the physical activity paradox within the context of WA and HRPL. We found that the effects of physical activity on WA and HRPL exhibited both similarities and differences. However, due to the cross-sectional nature of this study, we were unable to directly identify causal relationships. To confirm the causal association between physical activity and its impact on WA and HRPL, future longitudinal studies are recommended. This research would aid in the development of appropriate clinical guidelines for OPA and LTPA. Furthermore, we observed a large variance in OPA and LTPA, with values unevenly distributed. Consequently, the 95% CI of the GAM expanded with increases in OPA and LTPA. The criterion of 600-MET min/wk, used to categorize OPA and LTPA into high/low variables, was derived from the total physical activity recommended by the World Health Organization [12]. Importantly, however, this does not reflect separate cut-offs established for OPA and LTPA. Additionally, potential confounding factors such as duration of work, family responsibilities, and hobbies may influence the associations. However, due to the lack of this information in our survey data, we were unable to incorporate these factors into our analytical model. Future research should be conducted to determine the appropriate levels of OPA and LTPA for maintaining healthy and productive workers.

In conclusion, the findings of this study suggest a positive correlation between LTPA and WA, as well as labor productivity. In contrast, OPA appears to have a negative association, indicating a paradoxical effect between these types of physical activities. Furthermore, among older adults with physically demanding jobs, LTPA may negatively correlate with labor productivity. This suggests the need for advice tailored to the individual’s work situation and age. For instance, it is generally recommendable to participate in LTPA to improve WA and prevent HRPL. However, for older workers in physically demanding roles, it may be prudent to avoid excessive engagement in LTPA during non-working hours. We anticipate that future studies will provide additional evidence applicable to a variety of situations.

## Figures and Tables

**Figure f4-epih-45-e2023096:**
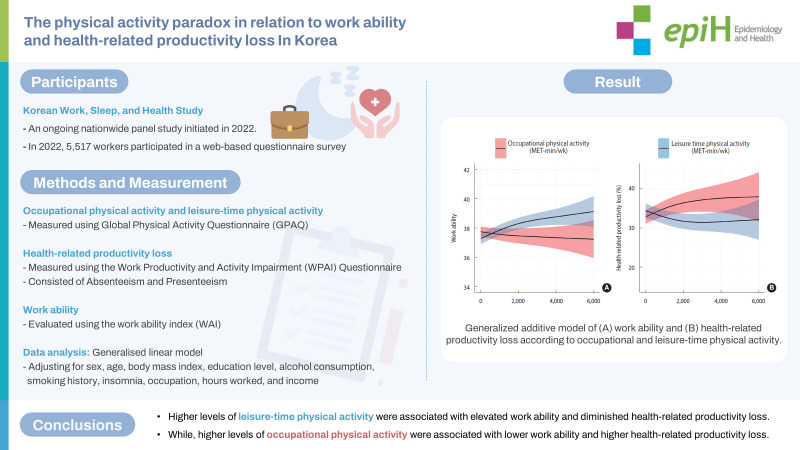


**Table 1.** LTPA, OPA, WA, and HRPL among study participants according to demographic characteristics

Characteristics	Total	LTPA (MET-min/wk)	OPA (MET-min/wk)	WA	HRPL (%)
Overall	5,501 (100)	788.02±1,307.31	369.75±1,451.91	37.64±5.43	29.26±25.89
Sex					
Male	3,000 (54.5)	912.53±1,408.06	507.51±1,724.66	37.96±5.44	29.11±25.72
Female	2,501 (45.5)	638.67±1,157.86	204.52±1,009.69	37.26±5.41	29.45±26.10
Age (yr)					
20-39	2,117 (38.5)	854.87±1,323.99	330.75±1,387.83	37.38±5.50	32.84±26.53
40-49	1,306 (23.7)	746.81±1,278.98	384.34±1,402.52	38.01±5.40	25.62±24.69
50-59	1,393 (25.3)	622.91±1,060.65	377.91±1,523.36	37.10±5.23	31.33±26.17
≥60	685 (12.5)	980.00±1,654.68	445.08±1,597.04	38.72±5.48	21.68±23.13
Education level (yr)					
≤12	859 (15.6)	793.82±1,452.15	555.40±2,008.20	37.79±5.47	26.62±25.28
>12	4,642 (84.4)	786.94±1,278.88	335.40±1,321.02	37.61±5.43	29.75±25.98
Occupation					
White-collar	3,718 (67.6)	790.72±1,275.14	200.54±845.57	37.80±5.28	29.48±25.93
Pink-collar	720 (13.1)	803.10±1,408.90	592.55±1,962.71	36.93±5.91	30.60±26.04
Blue-collar	1,063 (19.3)	768.34±1,347.48	810.70±2,340.58	37.58±5.60	27.61±25.59
Alcohol					
Low	4,643 (84.4)	795.19±1,322.39	360.88±1,389.02	37.61±5.46	29.52±25.96
High	858 (15.6)	749.23±1,222.49	417.76±1,753.73	37.80±5.31	27.89±25.50
Smoking					
Never	2,995 (54.5)	756.35±1,256.62	263.64±1,160.49	37.87±5.26	28.86±25.67
Ever smoker	2,506 (45.6)	825.86±1,364.82	496.58±1,728.94	37.37±5.62	29.75±26.16
Insomnia					
No	4,314 (78.4)	810.64±1,333.73	455.23±1,782.08	38.58±4.99	25.24±24.38
Yes	1,187 (21.6)	705.80±1,203.38	346.24±1,346.23	34.21±5.59	43.89±25.97
BMI (kg/m^2^)					
<25	3,696 (67.2)	788.08±1,313.52	359.32±1,430.72	37.80±5.40	29.14±25.89
≥25	1,805 (32.8)	787.90±1,294.87	391.13±1,494.53	37.31±5.49	29.53±25.90
Hours worked (hr/wk)					
<40	1,144 (20.8)	822.02±1,316.23	370.12±1,434.79	37.65±5.46	29.37±25.76
40-52	3,606 (65.6)	784.47±1,305.13	308.96±1,168.91	37.77±5.36	28.28±25.62
≥52	751 (13.7)	753.25±1,304.73	661.12±2,377.23	37.01±5.71	33.85±26.93
Income (KRW)^1^					
<2,500,000	1,926 (35.0)	685.09±1,220.82	354.66±1,498.88	36.98±5.61	29.48±26.24
<5,000,000	2,981 (54.2)	825.98±1,334.65	386.27±1,488.67	37.82±5.31	29.46±25.79
≥5,000,000	594 (10.8)	931.26±1,413.99	355.80±1,057.70	38.88±5.19	27.59±25.25

Values are presented as number (%) or mean±standard deviation. LTPA, leisure-time physical activity; OPA, occupational physical activity; WA, work ability; HRPL, health-related productivity loss; MET, metabolic equivalent; BMI, body mass index; KRW, Korean won.

1 Net monthly salary.

**Table 2.** Mean difference and 95% confidence interval of WA and HRPL according to OPA and LTPA^1^

Variables		Model 1	Model 2	Model 3
WA				
LTPA	Low	Reference	Reference	Reference
	High	1.50 (1.20, 1.81)	1.51 (1.21, 1.82)	1.10 (0.82, 1.38)
OPA	Low	Reference	Reference	Reference
	High	-1.20 (-1.62, -0.79)	-1.39 (-1.81, -0.98)	-0.90 (-1.29, -0.52)
HRPL (%)				
LTPA	Low	Reference	Reference	Reference
	High	-4.57 (-6.02, -3.11)	-5.02 (-6.46, -3.57)	-3.97 (-5.32, -2.62)
OPA	Low	Reference	Reference	Reference
	High	6.56 (4.58, 8.55)	7.18 (5.21, 9.16)	5.93 (4.07, 7.79)

Estimated using linear regression and compared to the reference (low LTPA or low OPA). WA, work ability; HRPL, health-related productivity loss; OPA, occupational physical activity; LTPA, leisure-time physical activity.

1 Model 1 was the crude model; Model 2 was adjusted for sex and age; and Model 3 was adjusted for sex, age, body mass index, education level, alcohol consumption, smoking status, insomnia, occupation, hours worked, and income.

**Table 3.** Mean difference and 95% confidence interval of WA and HRPL within each subgroup for OPA and LTPA^1^

Variables	Model 1	Model 2	Model 3
WA (OPA, LTPA)			
Low, High	Reference	Reference	Reference
Low, Low	-1.55 (-1.88, -1.22)	-1.55 (-1.88, -1.23)	-1.12 (-1.42, -0.82)
High, High	-1.32 (-1.83, -0.80)	-1.50 (-2.01, -0.98)	-0.95 (-1.42, -0.48)
High, Low	-2.55 (-3.27, -1.83)	-2.76 (-3.48, -2.04)	-1.94 (-2.60, -1.28)
HRPL (%) (OPA, LTPA)			
Low, High	Reference	Reference	Reference
Low, Low	5.02 (3.44, 6.59)	5.49 (3.93, 7.05)	4.31 (2.85, 5.76)
High, High	7.67 (5.20, 10.14)	8.34 (5.89, 10.79)	6.77 (4.47, 9.06)
High, Low	9.58 (6.13, 13.03)	10.58 (7.16, 13.99)	8.72 (5.51, 11.93)

Estimated using linear regression and compared to the reference (low LTPA or low OPA). WA, work ability; HRPL, health-related productivity loss; OPA, occupational physical activity; LTPA, leisure-time physical activity.

1 Model 1 was the crude model; Model 2 was adjusted for sex and age; and Model 3 was adjusted for sex, age, body mass index, education level, alcohol consumption, smoking status, insomnia, occupation, hours worked, and income.

**Table 4.** Mean difference and 95% confidence interval of WA and HRPL within each LTPA and OPA subgroup by age group^1^

Variables			Age (yr)	
			<60	≥60
WA	LTPA	Low	Reference	Reference
		High	1.104 (0.808, 1.399)	1.150 (0.332, 1.969)
	OPA	Low	Reference	Reference
		High	-0.789 (-1.202, -0.375)	-1.555 (-2.570, -0.540)
HRPL (%)	LTPA	Low	Reference	Reference
		High	-4.276 (-5.737, -2.815)	-1.979 (-5.497, 1.539)
	OPA	Low	Reference	Reference
		High	5.677 (3.631, 7.723)	7.406 (3.043, 11.768)

Estimated via linear regression and compared to the reference (low LTPA or low OPA). WA, work ability; HRPL, health-related productivity loss; LTPA, leisure-time physical activity; OPA, occupational physical activity.

1 The model was adjusted for sex, age, body mass index, education level, alcohol consumption, smoking history, insomnia, occupation, hours worked, and income.
